# The CXCR4-LASP1-eIF4F Axis Promotes Translation of Oncogenic Proteins in Triple-Negative Breast Cancer Cells

**DOI:** 10.3389/fonc.2019.00284

**Published:** 2019-04-24

**Authors:** Cory M. Howard, Nicole Bearss, Boopathi Subramaniyan, Augustus Tilley, Sangita Sridharan, Nancy Villa, Christopher S. Fraser, Dayanidhi Raman

**Affiliations:** ^1^Department of Cancer Biology, University of Toledo Health Science Campus, Toledo, OH, United States; ^2^Department of Molecular and Cellular Biology, University of California, Davis, Davis, CA, United States

**Keywords:** CXCR4, LASP1, eIF4F, eIF4A1, eIF4B, breast cancer, protein translation

## Abstract

Triple-negative breast cancer (TNBC) remains clinically challenging as effective targeted therapies are lacking. In addition, patient mortality mainly results from the metastasized lesions. CXCR4 has been identified to be one of the major chemokine receptors involved in breast cancer metastasis. Previously, our lab had identified LIM and SH3 Protein 1 (LASP1) to be a key mediator in CXCR4-driven invasion. To further investigate the role of LASP1 in this process, a proteomic screen was employed and identified a novel protein-protein interaction between LASP1 and components of eukaryotic initiation 4F complex (eIF4F). We hypothesized that activation of the CXCR4-LASP1-eIF4F axis may contribute to the preferential translation of oncogenic mRNAs leading to breast cancer progression and metastasis. To test this hypothesis, we first confirmed that the gene expression of CXCR4, LASP1, and eIF4A are upregulated in invasive breast cancer. Moreover, we demonstrate that LASP1 associated with eIF4A in a CXCL12-dependent manner via a proximity ligation assay. We then confirmed this finding, and the association of LASP1 with eIF4B via co-immunoprecipitation assays. Furthermore, we show that LASP1 can interact with eIF4A and eIF4B through a GST-pulldown approach. Activation of CXCR4 signaling increased the translation of oncoproteins downstream of eIF4A. Interestingly, genetic silencing of LASP1 interrupted the ability of eIF4A to translate oncogenic mRNAs into oncoproteins. This impaired ability of eIF4A was confirmed by a previously established 5′UTR luciferase reporter assay. Finally, lack of LASP1 sensitizes 231S cells to pharmacological inhibition of eIF4A by Rocaglamide A as evident through BIRC5 expression. Overall, our work identified the CXCR4-LASP1 axis to be a novel mediator in oncogenic protein translation. Thus, our axis of study represents a potential target for future TNBC therapies.

## Introduction

Breast cancer is the second leading cause of death due to cancer in women. One out of eight women (13%) will develop breast cancer in her lifetime (1). Mortality in breast cancer patients is mainly due to metastasis to the lungs, bone, and the brain. More specifically, triple-negative breast cancer (TNBC) is a devastating subtype with a low survival rate. Heterogeneity and plasticity observed in TNBC (2, 3) often results in chemoresistance, tumor relapse, and poor patient outcome. Therefore, it is imperative to find novel (and effective) targets for patients diagnosed with TNBC.

One potential approach to target TNBC cells has been through the inhibition of various chemokine receptors. Overall, this group of proteins plays an essential role in the tumor microenvironment to facilitate breast cancer progression and metastasis (4–10). More specifically, the CXCL12-CXCR4 signaling pathway has been associated with TNBC invasiveness and chemotactic homing (4, 6, 11–15). Previously, we reported that the C-terminal tail of CXCR4 directly binds to LIM and SH3 protein 1 (LASP1) (16) and knock down of LASP1 ablated CXCR4-driven invasion (17). LASP1 is an adaptor protein that has been shown to mediate cell migration, proliferation, and survival in several breast cancer cell lines (16–20). Additionally, LASP1 dissociates from the CXCR4 C-terminal tail upon CXCL12 stimulation (16). We therefore hypothesized that stimulation with CXCL12 could promote LASP1 to modulate the signaling network of CXCR4 via transient protein-protein interactions. Subsequently, we performed a proteomic screen for novel LASP1 interacting proteins (17). Eukaryotic initiation factors 4A and 4B (eIF4A and eIF4B) were identified to be interacting proteins. Both eIF4A and eIF4B are essential components of the eukaryotic initiation factor 4F complex (eIF4F).

The eIF4F complex consists of three core subunits: eIF4E, the cap binding subunit; eIF4A, an RNA helicase; and eIF4G1, a large scaffolding protein. Ultimately, selection of an mRNA by the eIF4F complex prepares it for successful recruitment of the 43S pre-initiation complex, and eventual ribosome assembly (21–28). More specifically, eIF4A catalyzes the ATP-dependent unwinding of RNA duplexes and requires the direct binding of its co-factor, eIF4B, along with eIF4G1, for its optimal activity (29–34). The eIF4F complex has been previously identified to be essential for the initiation and maintenance of a malignant phenotype in human mammary epithelial cells (35). Suppression of eIF4F can also affect the maintenance, progression, and metastasis of breast cancer in *in vivo* models (36–38). Elevated protein expression levels of eIF4A (39) and eIF4B have been observed in breast cancer patients (40). Moreover, eIF4A, eIF4B, and eIF4E were all found to be independent predictors of poor outcome in ER-negative breast cancer (40).

The current notion within the field is that the eIF4F complex has been identified to be a critical node of cancer biology due to many oncogenic mRNAs containing secondary structures within their 5′untranslated regions (5′UTRs) (41). Thus, cancer cells preferentially rely on eIF4A to unwind these structured 5′UTRs or stem-loop structures (SLS). Without eIF4F complex formation and activity, the secondary structure of the 5′UTR would stall ribosome scanning and detection of the methionine start codon (AUG) (42, 43). As a result, many oncogenic proteins would remain at steady-state levels and this would hinder malignancy. Several of these SLS-containing oncogenic mRNAs include: BIRC5 (Survivin), Cyclin D1 (CCND1), Ornithine Decarboxylase (ODC), Murine Double Minute 2 (Mdm2), Rho A kinase1 (ROCK1), Mucin-1C (MUC-1C), Sin1, and ADP Ribosylation Factor 6 (ARF6) (22, 25, 28, 44–46). In this paper, we pursued BIRC5, CCND1, ROCK1, and Mdm2 as eIF4A-dependent target genes.

Additionally, we were also interested in the influence of CXCR4 on the eIF4F complex through G-protein coupled receptor signaling. CXCR4 has been previously shown to activate both ribosomal S6 kinases: p90 ribosomal S6 kinase (p90^rsk^–via the ERK pathway) (47) and p70-S6 kinase (p70^rsk^–via the mTORC1 pathway) (48). These two major kinases have been established to feed into cap-dependent mRNA translation through modulation of regulatory proteins such as 4E-BP1 (49, 50). In its phosphorylated form, 4E-BP1 releases eIF4E to promote eIF4F complex formation. In addition, eIF4B is specifically phosphorylated on Ser422 by p90^rsk^ and p70^rsk^ kinases. This phosphorylated form of eIF4B is reported to increase the rate of translation (51, 52). Finally, active p70^rsk^ and p90^rsk^ also induces the phosphorylation and degradation of the tumor suppressor, programmed cell death protein 4 (PDCD4), an endogenous inhibitor of eIF4A (53). Despite strong primary evidence on several signaling pathways feeding into the eIF4F complex, limited literature exists on the phosphorylation status of these proteins following activation of CXCR4.

In this study, we confirm our initial findings from the proteomic screen and demonstrate that LASP1 can interact with both eIF4A and eIF4B. Importantly, the LASP1-eIF4A and LASP1-eIF4B interaction is shown to be CXCL12-dependent. In addition, the ability of CXCR4 to impact the phosphorylation of eIF4F regulatory proteins is provided. Taken together, we hypothesize that activation of CXCR4 can promote eIF4F complex formation and activity through LASP1 and cell signaling. As a result, the translation of oncogenic proteins is promoted thereby mediating an invasive and metastatic phenotype commonly associated with CXCR4.

## Materials and Methods

### Bioinformatics Analysis

To determine the significance of the CXCR4-LASP1-eIF4A/B axis in patient tissues, gene expression data was obtained and analyzed using Oncomine™ (54–56). Settings in the program were limited to a “cancer vs. normal analysis” and “breast cancer.” Data from two representative datasets are shown. Datasets include: Radvanyi Breast (PNAS, 08/02/2005) and TCGA Breast (The Cancer Genome Atlas, 09/02/2011). Box and whisker plots of the log2 median centered ratio for each cancer subtype were generated in the “R” statistical package (version 3.5.1) and the generated graphics were modified in Inkscape (version 0.92.3).

### Cell Culture

MDA-MB-231 human breast cancer cells (MDA-MB-231: ATCC® HTB-26™, Manassas, VA) were previously sorted for high cell surface expression of CXCR4 (denoted as 231S cells) and are described elsewhere (17). *293*-HA-CXC*R4* cells: Human embryonic kidney 293 cells (HEK-293: ATCC® CRL-1573™, Manassas, VA) stably expressing human CXCR4 are also described previously (17). MC*F7* series: MCF7 breast cancer cells (MCF7: ATCC® HTB-22™, Manassas, VA) expressing empty vector, wild-type CXCR4 (wild-type), or CXCR4 with a truncated C-terminal domain (ΔCTD) were characterized previously (8). Cells were maintained in Dulbecco's modified eagle medium (DMEM) supplemented with 4 mM L-glutamine, +4,500 mg/L glucose, sodium pyruvate (GE Healthcare Life Sciences, Pittsburgh, PA, Cat. No. SH30243.01), 10% heat-inactivated fetal bovine serum (FBS) (Denville Scientific, Swedesboro, NJ, Cat. No. FB5001-H), and Penicillin (100 I.U.)/Streptomycin (100 μg/ml) (Corning, Corning, NY, Cat. No. 30-002-CI).

### Generation of LASP1 Knockdown and Knockout Cell Lines

LASP1 was stably knocked down (KD) in 231S cells using shRNA constructs (V2LHS_64685 and V2LHS_64686, Open Biosystems, Huntsville, AL) (17). A non-silencing (NS) shRNA served as the wild type control (denoted as 231S LASP1 NS and KD). In order to obtain a genetic knockout (KO) of LASP1, LASP1 CRISPR/Cas9 knockout plasmids were purchased (Santa Cruz, Dallas, TX, Cat. No. sc-404630). Cells were transfected using UltraCruz reagent (Santa Cruz, Dallas, TX, Cat. No. sc-395739) according to the manufacturer's instructions. The supernatant was removed 24 h later and replaced with complete media. Cells were further cultured for 72 h post-transfection. Subsequently, LASP1-KO cells were sorted for GFP and single KO cells were isolated by limiting dilution. KO of LASP1 was confirmed by Western blotting. Non-targeting CRISPR/Cas9 plasmids served as the control (Santa Cruz, Dallas, TX, Cat. No. sc-418922). These plasmids encode the Cas9 nuclease and non-specific 20 nucleotide guide RNAs (denoted CRISPR control and LASP1 KO).

### Co-immunoprecipitation Assay

231S cells were serum-starved for 1 h and stimulated with 10–20 nM CXCL12 (PeproTech, Rocky Hill, NJ, Cat. No. 300-28A) over different time points. Total cell lysates were prepared by lysing the cells in co-immunoprecipitation buffer (Co-IP buffer) (50 mM Tris pH 8.0, 100 mM NaCl, 0.1% IGEPAL CA-630, 0.1% Deoxycholate and 5 mM EDTA) supplemented with protease inhibitor cocktail (Sigma-Aldrich, St. Louis, MO, Cat. No. P8340-5ML), phosphatase inhibitor cocktail 2 (Sigma-Aldrich, St. Louis, MO, Cat. No. P5726), and phosphatase inhibitor cocktail 3 (Sigma-Aldrich, St. Louis, MO, Cat. No. P0044) for 10 min at 4°C. Total protein in the clarified lysate was quantified using a Bradford protein assay (Bio-Rad, Hercules, CA, Cat. No. 5000006). 1 mg of total protein lysate was employed in all immunoprecipitation reactions. eIF4B was immunoprecipitated by using 2 μg of eIF4B antibody (Cell Signaling Technology, Danvers, MA, Cat. No. 13088). Mouse (G3A1) mAb IgG1 Isotype Control (Cell Signaling Technology, Danvers, MA, Cat. No. 5415) served as the mock control. Next, eIF4A was immunoprecipitated using 2 μg of eIF4A1 antibody (Cohesion Biosciences, London, Purley, Cat. No. CQA1180). His-Tag (D3L10) XP® Rabbit mAb (Cell Signaling Technology, Danvers, MA, Cat. No. 12698) was employed for the mock condition. Finally, in the reciprocal Co-IP, LASP1 was immunoprecipitated by using 2 μg LASP1 antibody (Biolegend, San Diego, CA, Cat. No. 909301). As in the eIF4B Co-IP, mouse (G3A1) IgG1 Isotype mAb was employed as the mock control. Prior to immunoprecipitation, lysates were pre-cleared with 20 μL of PureProteome™ Protein G Magnetic Beads (Millipore, Billerica, MA, Cat No. LSKMAGG10) for 2 h at 4°C. Immunoprecipitation reactions were then allowed to proceed with 20 μL of protein G magnetic beads and the appropriate amount of antibody for 2 h at 4°C. Beads were washed with Co-IP buffer. To avoid heavy chain contamination (55 kDa) in the eIF4A Co-IP, antibodies were cross-linked using BS^3^ (according to Millipore recommendations). Proteins of interest were analyzed by sodium dodecyl sulfate-polyacrylamide gel electrophoresis (SDS-PAGE) and Western blotting.

### m^7^-GTP Pull-Down Assay

231S cells were serum starved for 1 h and cells were stimulated (and prepared) as described in the co-immunoprecipitation section. 100 nM AMD3465 (CXCR4 antagonist, Sigma-Aldrich, St. Louis, MO, Cat. No. SML1433-5MG) was incubated 30 min prior to stimulation. 1 mg of total protein lysate in 1 mL of Co-IP buffer was incubated with 25 μL of m^7^-GTP agarose beads overnight at 4°C (Jena Biosciences, Jena, Germany, Cat. No. AC-155S). Following incubation, beads were washed with Co-IP buffer. Protein was eluted and analyzed by SDS-PAGE and Western blotting.

### GST-LASP1 Pull-Down of eIF4A and eIF4B

The open reading frame of the human LASP1 gene (Open Biosystems, Huntsville, AL) was engineered with BamHI and XhoI cloning sites using the following gene specific primers: 5′-CTAGCTGGATCCATGAACC CCAACTGCGCC-3′ (forward), and 5′-CTAGCTCTCGAGTCAGATGGCCTCCACGTA-3′ (reverse). Following amplification by polymerase chain reaction (PCR), LASP1 was inserted into the GST bacterial expression vector pGEX-6P-1 (GE Healthcare Life Sciences, Pittsburgh, PA, Cat. No. 28954648). The verity of the DNA construct was confirmed by sequencing (Eurofins Genomics, Louisville, KY). Both the GST-LASP1 and empty pGEX-6P-1 vector (GST control) were transformed into E. coli BL21 strain for the production of GST and GST-LASP1 proteins using standard protocols described elsewhere (20). For the pull-down assays, 1.5 nmoles of the GST control protein (40.5 μg) and GST-LASP1 (85.1 μg) bound to glutathione agarose beads (Thermo Scientific, Rockford, IL, Cat No. 16100) were equilibrated in Co-IP buffer and incubated with 1 mg of total protein lysates from 231S cells in 1 mL of Co-IP buffer for 2 h at 4°C. After washing the beads with Co-IP buffer, the bound proteins were eluted and analyzed by SDS-PAGE and Western blotting.

### Direct Binding of LASP1 to eIF4A and eIF4B

Recombinant eIF4A and eIF4B were purified to homogeneity according to previously published protocols (21, 27). In one set of direct binding experiments, 1.5 nmoles of GST-LASP1 and GST-control beads in 1 mL of Co-IP buffer were incubated with purified recombinant eIF4A and eIF4B overnight at 4°C. Beads were then washed with Co-IP buffer and bound protein was eluted by SDS-PAGE and Western blotting. To confirm that the binding is able to occur in a 1:1 molar ratio (and also in solution), GST-LASP1 and GST were eluted from the beads using glutathione elution buffer (10 mM L-glutathione, 50 mM Tris pH 8.0). Eluted protein was quantified using a Bradford protein assay. Equimolar amounts of proteins were incubated in a final volume of 1 mL of Co-IP buffer overnight at 4°C. Complexes were then re-captured with 10 μL glutathione agarose beads for 1 h at 4°C. Finally, beads were washed with Co-IP buffer and bound proteins were eluted and analyzed by SDS-PAGE and Western blotting. Purity of the recombinant proteins was confirmed by SDS-PAGE and staining with Imperial™ Protein Stain (Thermo Scientific, Rockford, IL, Cat No. 24615) (5 ng detection limit).

### Proximity Ligation Assay (PLA)

The Duolink *In Situ* Orange Fluorescent kit (Sigma/Aldrich, St. Louis, MO, Cat. No. DUO92102-1KT) was employed to detect the endogenous interaction between LASP1 and eIF4A *in situ* in 231S cells. The PLA was performed according to manufacturer's instructions using a rabbit eIF4A1 antibody (Cohesion Biosciences, London, Purley, Cat. No. CQA1180) and a mouse LASP1 antibody (Biolegend, San Diego, CA, Cat. No. 909301) (17). The single Ab control condition represents the PLA reaction with only the LASP1 antibody. In addition, cells were stained with phalloidin (Life Technologies, Carlsbad, CA, Cat. No. A12379) and nuclei with DRAQ5 (Thermo Scientific, Rockford, IL, Cat No. 62251). Cells were stimulated and inhibited as described elsewhere in the paper. Moreover, cells were fixed, stained, and permeabilized with standard methods (17). The images were acquired by two-photon confocal microscopy and processed with Leica Application Suite X software (Leica, Wetzlar, Germany). Quantification of the interaction dots was performed using ImageJ.

### Western Blotting

Cell lysates were prepared and quantified as described elsewhere. Western blots were incubated with the following 1°Abs: Cyclin D1 (Cell Signaling Technology, Danvers, MA, Cat. No. 2922), eIF4A C32B4 (Cell Signaling Technology, Danvers, MA, Cat. No. 2013), eIF4B 1F5 (Cell Signaling Technology, Danvers, MA, Cat. No. 13088), p-eIF4B S422 (Thermo Scientific, Rockford, IL, Cat No. PA5-38362), eIF4E C46H6 (Cell Signaling Technology, Danvers, MA, Cat. No. 2067), eIF4G C45A4 (Cell Signaling Technology, Danvers, MA, Cat. No. 2469), GST 26H1 (Cell Signaling Technology, Danvers, MA, Cat. No. 2624), LASP1 8C6 (Biolegend, San Diego, CA, Cat. No. 909301), MDM2 SMP14 (Santa Cruz Biotechnology, Dallas, TX, Cat. No. sc-965), PDCD4 D29C6 (Cell Signaling Technology, Danvers, MA, Cat. No. 9535), p-PDCD4 S67 (Sigma-Aldrich, St. Louis, MO, Cat. No. P0072), ROCK1 C8F7 (Cell Signaling Technology, Danvers, MA, Cat. No. 4035), Survivin 71G4B7 (Cell Signaling Technology, Danvers, MA, Cat. No. 2808), 4E-BP1 53H11 (Cell Signaling Technology, Danvers, MA, Cat. No. 9644), p-4E-BP1 Thr70 (Cell Signaling Technology, Danvers, MA, Cat. No. 9455), and β-tubulin D66 (Sigma/Aldrich, St. Louis, MO, Cat. No. T0198). Following primary incubation, Western blots were incubated with Goat anti-Mouse IgG (H + L) Superclonal™ Secondary Ab conjugated to HRP (Thermo Scientific, Rockford, IL, Cat No. A28177) or Goat anti-Rabbit IgG (H+L) Superclonal™ Secondary Ab conjugated to HRP (Thermo Scientific, Rockford, IL, Cat No. A27036). Finally, blots were developed with Amersham™ ECL™ Prime Western Blotting Detection Reagent (GE Healthcare Life Sciences, Pittsburgh, PA, Cat. No. RPN2232) and HyBlot ES™ Autoradiography Film (Denville Scientific, Swedesboro, NJ, Cat. No. E3212). Densitometry of the Western blots was performed using ImageJ. Calculation of fold change is given in the figure legend for each experiment.

### Real-Time PCR

Total RNA was extracted from 231S LASP1 NS and 231S LASP1 KD cells using an RNeasy® Mini Kit according to the manufacturer's instructions (Qiagen, Germantown, MD, Cat. No. 74104). Following RNA isolation, cDNA was synthesized using a SuperScript™ III First-Strand Synthesis Kit (Invitrogen, Carlsbad, CA, Cat. No. 18080-400). 2000 ng of input RNA and random hexamer primers were used according to the manufacturer's instructions. Finally, real-time PCR was performed using 2X PowerUp™ SYBR™ Green Master Mix (Applied Biosystems, Foster City, CA, Cat. No. A25741), 2 μL of cDNA, and 2 μL of the following forward and reverse primers (300 nM): ROCK1: 5′-AACATGCTGCTGGATAAATCTGG-3′ and 5′-TGTATCACATCGTACCATGCCT-3′ MDM2: 5′-CCTTCGTGAGAATTGGCTTC-3′ and 5′-CAACACATG ACTCTCTGGAATCA-3′ CCND1: 5′-ATGTTCGTGGCCTCTAAGATG A-3′ and 5′-CAGGTTCCACTTGA GCTTGTTC-3′ BIRC5: 5′-AAGAACTGGCCCTTCTTGGA-3′ and 5′-CAACCGGACGAATGCTTTT-3′ β-tubulin: 5′-TTGGCCAGATCTTTAGACCAGACAAC-3′ and 5′-CCGTACCACATCCAGGACAGAATC-3′. Real time data was analyzed using the ΔΔCt method with β-tubulin primers as the control. The values from 231S LASP1 NS cells were then set to 1.

### GQ 5′UTR Luciferase Assay

The GQ 5′UTR luciferase assay is a previously published method to assess the endogenous activity of eIF4A in cells (42). Four tandem repeats of the (CGG)_4_ 12-mer motif (GQ 5′UTR) or a random sequence matched for length and GC content (Random GQ 5′UTR) were cloned into the 5′UTR of firefly pGL4.10 luc2 (Promega, Madison, WI, Cat. No. E6651) containing the CMV promoter. To create these constructs, CMV was first cloned into pGL4.10 luc2 by employing KpnI and XhoI restriction sites. The CMV promoter was amplified from pcDNA3.0 (Invitrogen, Carlsbad, CA, Cat. No. V79020) using the following primers: 5′-TTTGTAGGTA CCGATGTACGGGCCAGATATAC-3′ and 5′-TTTGTACTCGAGGTATTAATTTCGATA AGC-3′. After successful insertion and verification of the CMV promoter, both 5′UTR sequences (GQ and Random GQ) were cloned before the luciferase open reading frame via added BglII and HindIII sites. This was accomplished with the following annealed oligonucleotides obtained commercially (Eurofins Genomics, Louisville, KY): GQ 5′UTR: 5′-GATCTCTAGGTTGAAAGTACTTTGACGGCGGCGGCGGTCAATCTTACGGCGGCGGCGGACATAGATACGGCGGCGGCGGTAGAAACTACGGCGGCGGCGGATTAGAATAGTAAAA-3′ and 5′-AGCTTTTTACTATTCTAATCCGCCGCCGCCGTAGTTTCTACCGCCGCCGCCGTATCTATGTCCGCCGCCGCCGTAAGATTGACCGCCGCCGCCGTCAAAGTACTTTCAACCTAGA-3′ Random GQ 5′UTR: 5′-GATCTCTAGGGCGCACGTACTTCGACAACGTCAGCGTTCAGCGTTCCAACGTCAGCGTACAGCGATCCAACGTCAGCGTTCTGCGCTACAACGTCAGCGTATCCGCGTAGCACAA-3′ and 5′-AGCTTTGTGCTACGCGGATACGCTGACGTTGTAGCGCAGAACGCTGACGTTGGATCGCTGTACGCTGACGTTGGAACGCTGAACGCTGACGTTGTCGAAGTACGTGCGCCCTAGA-3′. 40 ng of each firefly luciferase construct was transfected along with 40 ng of pGL4.74 hRluc (Promega, Madison, WI, Cat. No. E6921). Following transfection, cells were incubated in serum free media overnight. Cells were lysed, protein lysates were then collected the next day. Firefly and renilla luciferase activity were assessed by employing the Dual-Luciferase® Reporter Assay System (Promega, Madison, WI, Cat. No. E1910). Data reflects firefly luciferase activity normalized to renilla readings with the CMV-pGL4.10 luc2 set to 1 for each cell type.

### Pharmacological Inhibition of eIF4A in 231S LASP1 NS and KD Cells

231S LASP1 NS/KD cells were plated into 96-well dishes (3,000 cells/well) and incubated with various amounts of Rocaglamide A (RocA) (Sigma/Aldrich, St. Louis, MO, Cat. No. SML0656) in low serum media (LSM-DMEM/0.5% FBS). Images were acquired via an IncuCyte® S3 Live-Cell Analysis System (Essen BioScience, Ann Arbor, Michigan). Two images per well were acquired every 2 h. Data was processed on the IncuCyte S3 software (Essen BioScience, Ann Arbor, Michigan). Only cells with an area >150 μm^2^ were analyzed to avoid cellular debris. Data is reflective of the percent confluence of each image at 36 h RocA incubation. Percent inhibition was calculated in reference to the DMSO control. The readings from 231S LASP1 NS cells were set to 1. In the cell viability experiments, 231S LASP1 NS/KD cells were seeded at 6,000 cells/well and allowed to attach and spread overnight. The following day, complete media was replaced with LSM and RocA. Cell viability was then determined 36 h later using a cell counting kit-8 (Sigma/Aldrich, St. Louis, MO, Cat. No 96992) according to the manufacturer's instructions. Data is reflective of the absorbance at 450 nm (A450). The DMSO controls were set to 100%.

### Statistical Analysis and Graph Preparation

Data analysis was performed using the R statistical program (version 3.5.1). Statistical significance between groups was determined by unpaired Student's *t*-tests with a “*p*”-value set to <0.05. Graphs were first generated in R and then modified in Inkscape (version 0.92.3).

## Results

### Breast Cancer Patient Samples Contain Elevated Levels of CXCR4, LASP1, eIF4A, eIF4B, and the Downstream Targets of eIF4A

**Figure 1:**
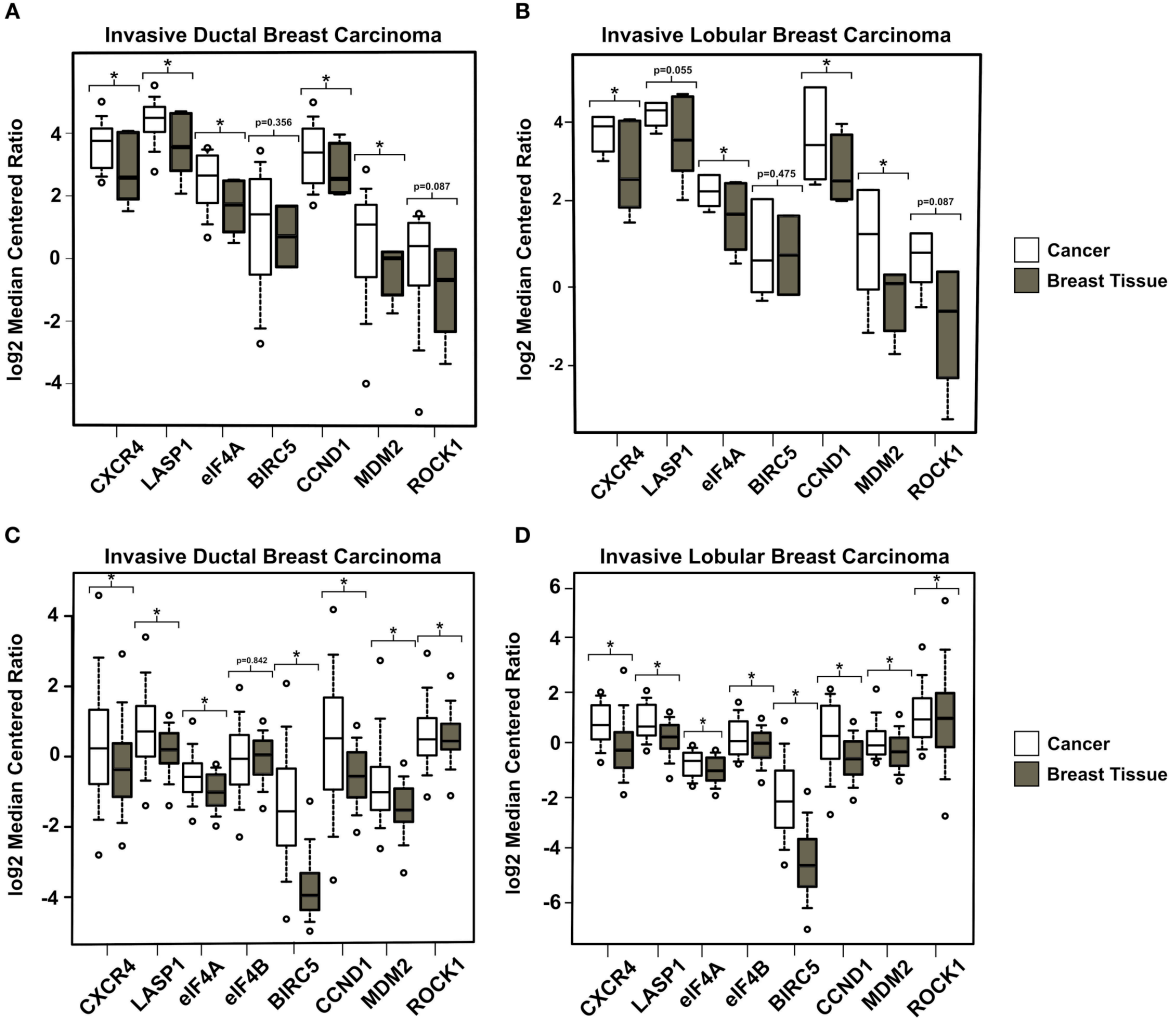
The CXCR4-LASP1-eIF4A/B Axis is Upregulated in Breast Carcinoma Patients. Gene expression data was obtained and analyzed using Oncomine.com. Two representative datasets were selected. Box and whisker plots of the log2 median centered ratio (fold change) are shown for each. **(A)** Radvanyi Breast Invasive Ductal Carcinoma (*n* = 31 for CXCR4, LASP1, eIF4A, and CCND1. *n* = 28 for BIRC5 and ROCK1. *n* = 27 for MDM2) vs. Breast Tissue (*n* = 9 for CXCR4, LASP1, eIF4A, and CCND1. *n* = 2 for BIRC5. *n* = 5 for MDM2 and ROCK1). **(B)** Radvanyi Breast Invasive Lobular Carcinoma (*n* = 7 for CXCR4, LASP1, eIF4A, and CCND1. *n* = 2 for BIRC5. *n* = 6 for MDM2 and ROCK1) vs. Breast Tissue (*n* = 9 for CXCR4, LASP1, eIF4A, and CCND1. *n* = 2 for BIRC5. *n* = 5 for MDM2 and ROCK1). **(C)** TCGA Breast Invasive Ductal Carcinoma (*n* = 389) vs. Breast Tissue (*n* = 61). **(D)** TCGA Breast Invasive Lobular Carcinoma (*n* = 36) vs. Breast Tissue (*n* = 61). ^*^ Indicates *p* < 0.05 as evaluated by student's *t*-tests.

In order to evaluate the gene expression profile of CXCR4, LASP1, eIF4A, and eIF4B in breast cancer patients, we analyzed breast cancer data sets using “Oncomine.” This online resource is a cancer microarray database and an integrated data-mining platform (54–56). Differential expression analyses were performed on “The Cancer Genome Atlas” (TCGA) and Radvanyi data sets. In each analysis, the gene expression profile of normal breast tissue was compared with invasive lobular carcinoma (ILC) and invasive ductal carcinoma (IDC) samples. We observed a significant elevation of gene expression for CXCR4, LASP1, eIF4A, and eIF4B. In addition, the genes downstream of eIF4A (BIRC5, CCND1, ROCK1, and MDM2) were also observed to have elevated expression levels (*p* < 0.05) (Figures 1A–D). Overall, this points to the clinical significance of our axis of study. Elevated mRNA levels of these genes established the premise that these proteins may play a vital role in oncoprotein translation to promote metastatic breast cancer. However, we recognize that future studies will need to confirm these findings at the protein level.

### LASP1 Associates With eIF4A Endogenously in a CXCL12-Dependent Manner *in situ*

**Figure 2:**
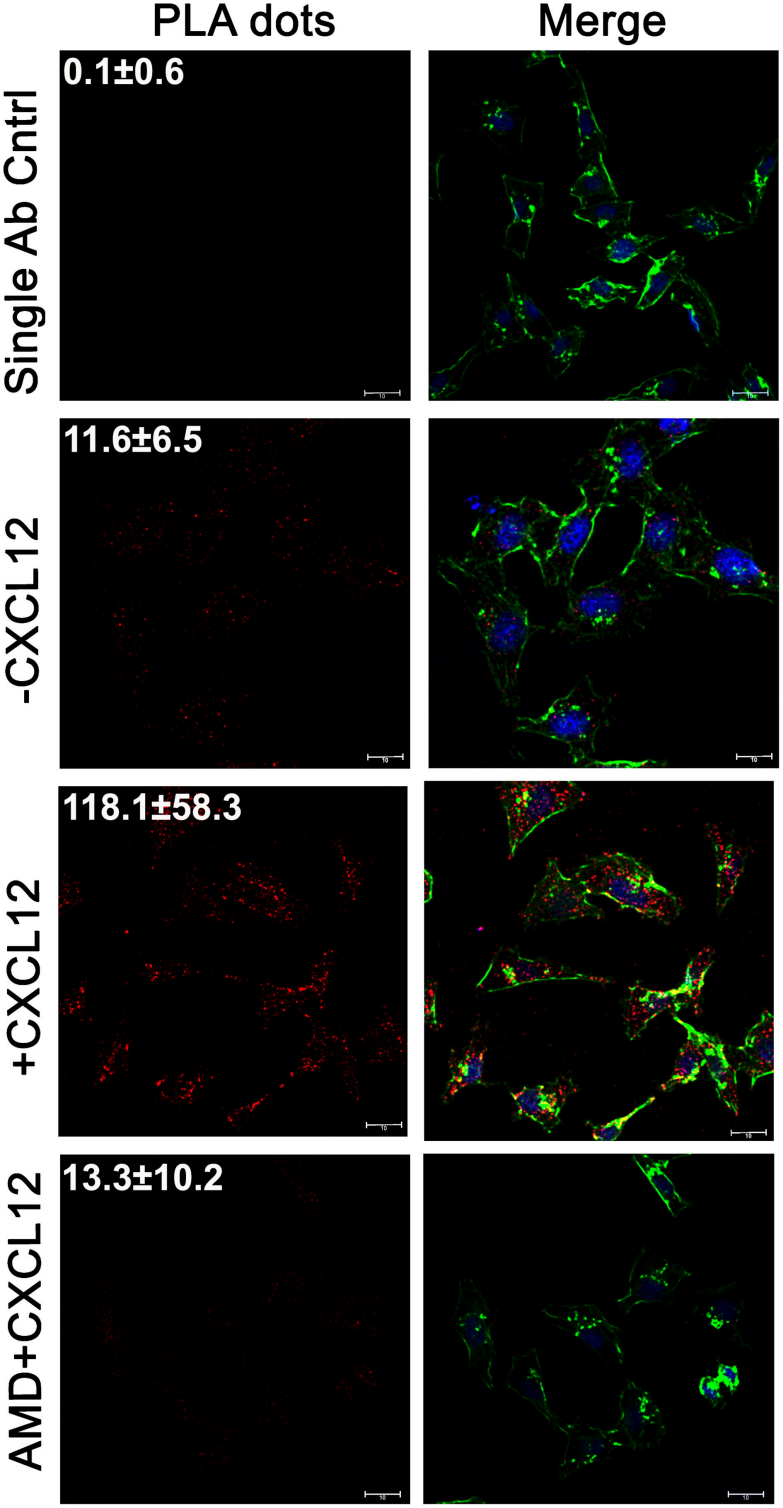
The LASP1-eIF4A interaction increases with CXCL12 stimulation *in situ*. The Proximity Ligation Assay (PLA) was used to visual the *in situ* interaction between LASP1 and eIF4A in 231S cells. Cells were stimulated with 20 nM CXCL12 for 5 min. 100 nM AMD3465 was added 30 min prior to stimulation. The single antibody control employs the PLA reaction using only the LASP1 antibody. Representative images of the PLA experiment are provided. Quantification indicates the average number of interactions/cell across multiple independent fields. (Single Ab Control: *n* = 39, -CXCL12: *n* = 46, +CXCL12: *n* = 29, +CXCL12/AMD3465: *n* = 15); Red-LASP1-eIF4A interaction, Green-phalloidin (actin), and Blue-DRAQ5 (nucleus); Scale bar−10 μm.

To initially confirm the LASP1-eIF4A interaction, we examined whether LASP1 would associate with eIF4A endogenously in 231S cells *in situ*. Following stimulation or inhibition of CXCR4, the endogenous interaction between LASP1 and eIF4A was probed by a proximity ligation assay (PLA). The single antibody control displayed almost no interaction dots (0.09 ± 0.6 dots—from 39 cells). In the unstimulated state (-CXCL12), a basal interaction was detected between LASP1 and eIF4A (11.6 ± 6.5 dots—from 46 cells). With CXCL12 incubation for 5 min, there was a 3-fold stimulation of the interaction between LASP1 and eIF4A (118 ± 58.3 dots—from 29 cells). The CXCL12-stimulated interaction between LASP1 and eIF4A can be abrogated to the basal level by the CXCR4 antagonist, AMD3465 (13.3 ± 10.2 dots—from 15 cells) (Figure 2).

### LASP1 Co-immunoprecipitates With eIF4A and eIF4B Endogenously in a CXCL12-Dependent Manner

**Figure 3:**
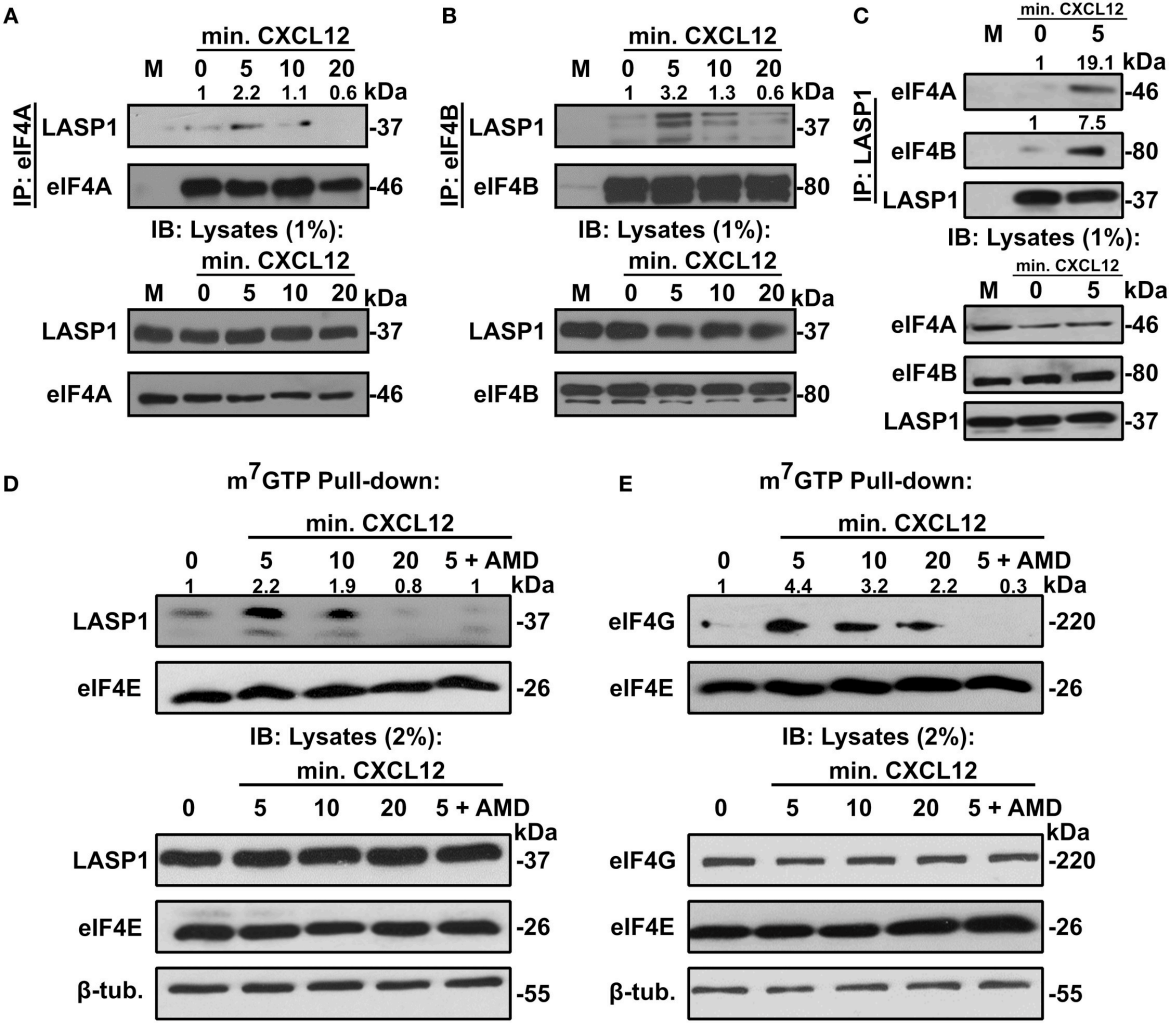
LASP1 interacts with the eIF4F complex in a CXCL12-dependent manner. **(A)** Co-immunoprecipitation assay of eIF4A and LASP1 in 231S cells following stimulation with 20 nM CXCL12 (*n* = 2). **(B)** Co-immunoprecipitation assay of eIF4B and LASP1 in 231S cells following stimulation with 10 nM CXCL12 (*n* = 3). **(C)** Co-immunoprecipitation assay of LASP1 and eIF4A/B following stimulation with 20 nM CXCL12 in 231S cells (*n* = 2). Fold change was calculated based off the densitometry ratio of co-immunoprecipitated/immunoprecipitated protein signal with 0 min. set to 1. **(D–E)** m^7^GTP pulldown assay in 231S cells following stimulation with 20 nM CXCL12 and 100 nM AMD3465 examining the interaction between: **(D)** LASP1-eIF4E (*n* = 3) and **(E)** eIF4G-eIF4E (*n* = 3). Fold change was calculated based off the densitometry ratio of co-precipitate (LASP1 or eIF4G)/precipitate (eIF4E) protein signal with 0 min. set to 1.

In order to further evaluate the association between LASP1, eIF4A1, and eIF4B, we performed co-immunoprecipitation assays with and without CXCL12 stimulation in 231S cells. In each of these co-immunoprecipitation experiments, both eIF4A and eIF4B associated with LASP1 endogenously in a CXCL12-dependent manner with peak interaction at 5 min (Figures 3A,B). To further validate the interaction, we also immunoprecipitated LASP1 and probed for the presence of eIF4A and eIF4B in the reciprocal Co-IP. There was a slight basal association of endogenous LASP1 and eIF4A1 which increased to 19.1-fold upon stimulation of CXCR4 for 5 min. Similarly, there was a 7.5-fold increase in association of LASP1 with eIF4B upon activation of CXCR4 (Figure 3C).

### Endogenous LASP1 Associates With the eIF4F Complex in a CXCL12-Dependent Manner

We next asked if LASP1 could be incorporated into the eIF4F complex upon stimulation of CXCR4. To address this question, we employed the previously established m^7^GTP-pulldown assay. Activation of CXCR4 in 231S cells resulted in an increased association of endogenous LASP1 with eIF4E. This association peaked at 5 (2.2-fold) min before returning a to basal level at 20 min. Importantly, the CXCL12-dependent recruitment of LASP1 at 5 min can be abrogated by pre-treatment of the cells with AMD3465 (Figure 3D). Furthermore, the eIF4G-eIF4E interaction has been commonly accepted to give an indication of eIF4F complex formation (57). As such, we explored the influence of CXCR4 on the eIF4G-eIF4E interaction. eIF4G was incorporated in a CXCL12-dependent manner with a peak recruitment at 5 min (4.4-fold) and slowly declined to 2.2-fold at 20 min, well above the baseline level. This peak recruitment at 5 min of CXCL12 treatment can also be reduced by AMD3465 (Figure 3E).

### LASP1 Directly Binds to Both eIF4A and eIF4B

**Figure 4:**
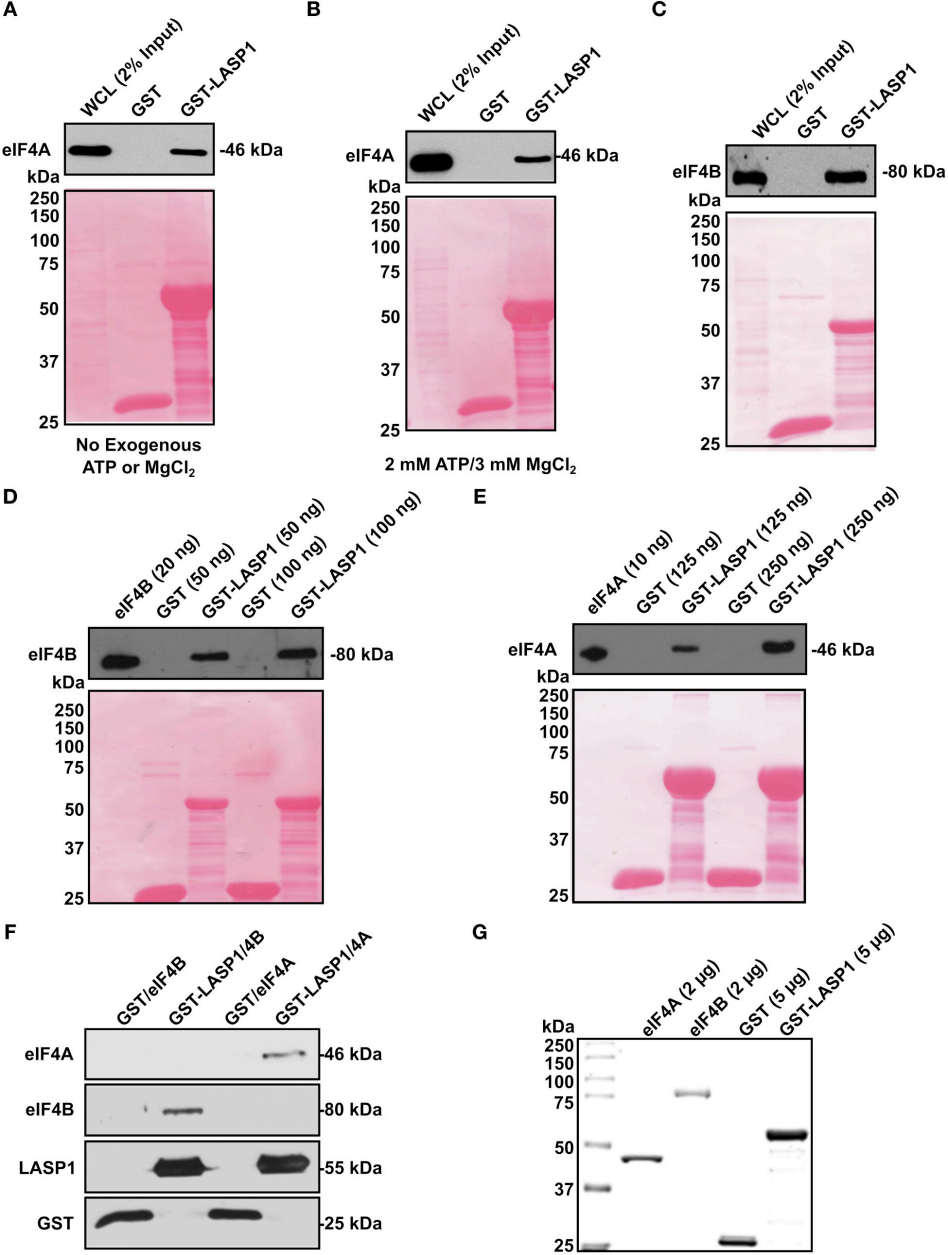
LASP1 directly interacts with both eIF4A and eIF4B. **(A–C)** 1 mg of 231S lysate was incubated with 1.5 nmoles of GST or GST-LASP1. **(A)** Presence of eIF4A was detected by Western blotting (*n* = 3). **(B)** 2 mM ATP and 3mM MgCl_2_ were exogenously added to the 231S lysate. Presence of eIF4A was then detected by Western blotting (*n* = 3). **(C)** Presence of eIF4B was detected by Western blotting (*n* = 3). **(D–E)** Purified eIF4A or eIF4B was incubated with 1.5 nmoles GST or GST-LASP1. Amounts of purified proteins are indicated in parenthesis. **(D)** Presence of eIF4B was detected by Western blotting (*n* = 3). **(E)** Presence of eIF4A was detected for by Western blotting (*n* = 3). Ponceau S stains of each blot are shown below to confirm loading of GST or GST-LASP1 following the elution from glutathione agarose beads. **(F)** Purified eIF4A and eIF4B were mixed with purified GST or GST-LASP1 in an equimolar ratio and in solution. Proteins complexes were then captured with glutathione beads and detected for by Western blotting (*n* = 1). **(G)** Imperial Protein Stain of purified eIF4A, eIF4B, GST, and GST-LASP1 (*n* = 1).

To further prove the interaction of LASP1 with eIF4A and eIF4B, we employed a GST-pulldown assay. In the eIF4A pulldown, we also added exogenous ATP (2 mM) and MgCl_2_ (3 mM) since eIF4A is an ATP-dependent enzyme. LASP1 associated robustly with eIF4A regardless of any exogenous addition of ATP and MgCl_2_ (Figures 4A,B). In addition, LASP1 robustly associated with eIF4B as well (Figure 4C). This experimental system was also used to test if LASP1 could directly bind to both eIF4A and eIF4B. Previous interaction experiments were unable to distinguish a direct interaction between these proteins. LASP1 directly bound to both purified, recombinant eIF4B and eIF4A in a dose-dependent manner (Figures 4D,E). Finally, to further confirm the validity of the direct binding experiments, we mixed equimolar amounts of purified GST-LASP1 with eIF4A or eIF4B in a solution and captured the complex with glutathione beads. As expected, LASP1 directly bound to both eIF4A and eIF4B (Figure 4F). The purity of the proteins employed in these experiments is shown (Figure 4G).

### Activation of CXCR4 Promotes Phosphorylation of PDCD4, eIF4B, and 4E-BP1

**Figure 5:**
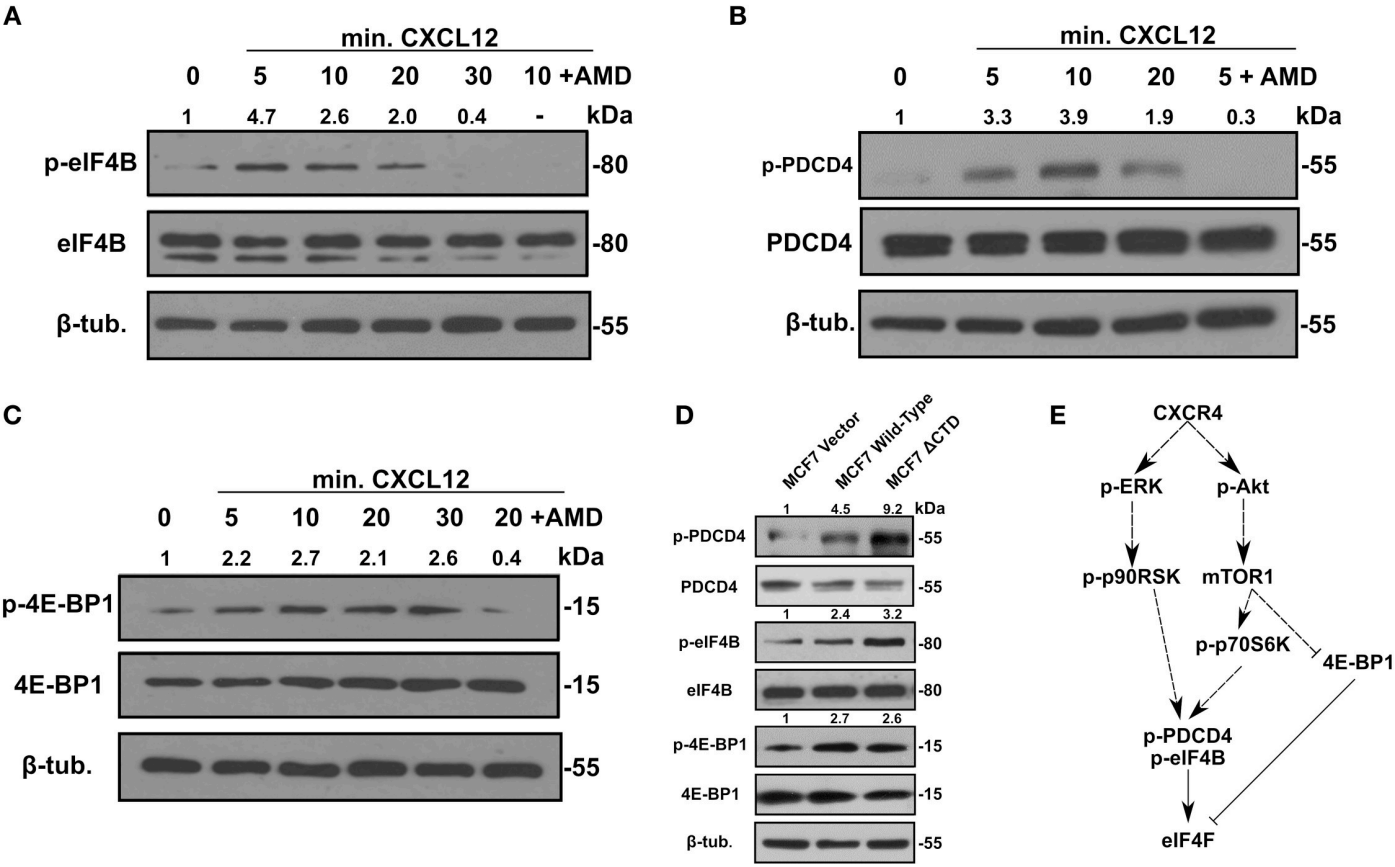
Activation of CXCR4 promotes phosphorylation of eIF4B, 4E-BP1, and PDCD4. 231S cells were stimulated with 10–20 nM CXCL12 for the indicated period. Phosphorylation status of **(A)** *p*-eIF4B S422 **(B)** p-PDCD4 S67 and **(C)** p-4E-BP1 Thr70 was determined by Western blotting (*n* = 3). Fold change indicates the densitometry ratio of (phospho-protein/total protein)/β-tubulin signal with 0 min. set to 1. **(D)** Status of *p*-eIF4B, *p*-PDCD4 S67, and *p*-4E-BP1 Thr70 in MCF7 vector, wild-type CXCR4, and CXCR4 ΔCTD cells (*n* = 3). Fold change indicates the densitometry ratio of (phospho-protein/total protein)/β-tubulin signal with MCF7 vector cells set to 1. **(E)** Proposed model of CXCR4 signaling and its effects on the eIF4F complex.

Aside from the influence of LASP1 on eIF4A and eIF4B, we next addressed whether activation of CXCR4 would feed into the activation of eIF4F complex through cell signaling. Interestingly, activation of CXCR4 led to phosphorylation of eIF4B on S422 by 5 min before declining (Figure 5A). The increase at 10 min in p-eIF4B levels can be abrogated with AMD3465. Next, we addressed if CXCR4 could promote the phosphorylation of PDCD4. With CXCR4 activation, the phosphorylation increased to 3.3-fold at 5 min and peaked at 10 min (3.9-fold). The increase at 5 min in pS67-PDCD4 level can be abrogated by pretreatment of cells with AMD3465 (Figure 5B). We then examined phosphorylation of 4E-BP1, which releases eIF4E and allows its incorporation into the eIF4F complex. Upon activation of CXCR4, pT70-4E-BP1 levels increased by 5 min and this effect could be abrogated with AMD3465 (Figure 5C). Finally, to further prove these findings, we utilized a MCF7-CXCR4 cell series which contains increasing basal activity of CXCR4 (8). As expected, MCF7-CXCR4 wild-type and ΔCTD cells had increases in levels of p-4E-BP1, p-PDCD4 and p-eIF4B over that of the vector control (Figure 5D). Based off of these findings and previous work done by others, the proposed model of CXCR4 signaling and its effects on the eIF4F complex is provided (Figure 5E).

### Activation of the CXCR4-LASP1 Axis Enhances Selective Expression of Genes Downstream of eIF4A

**Figure 6:**
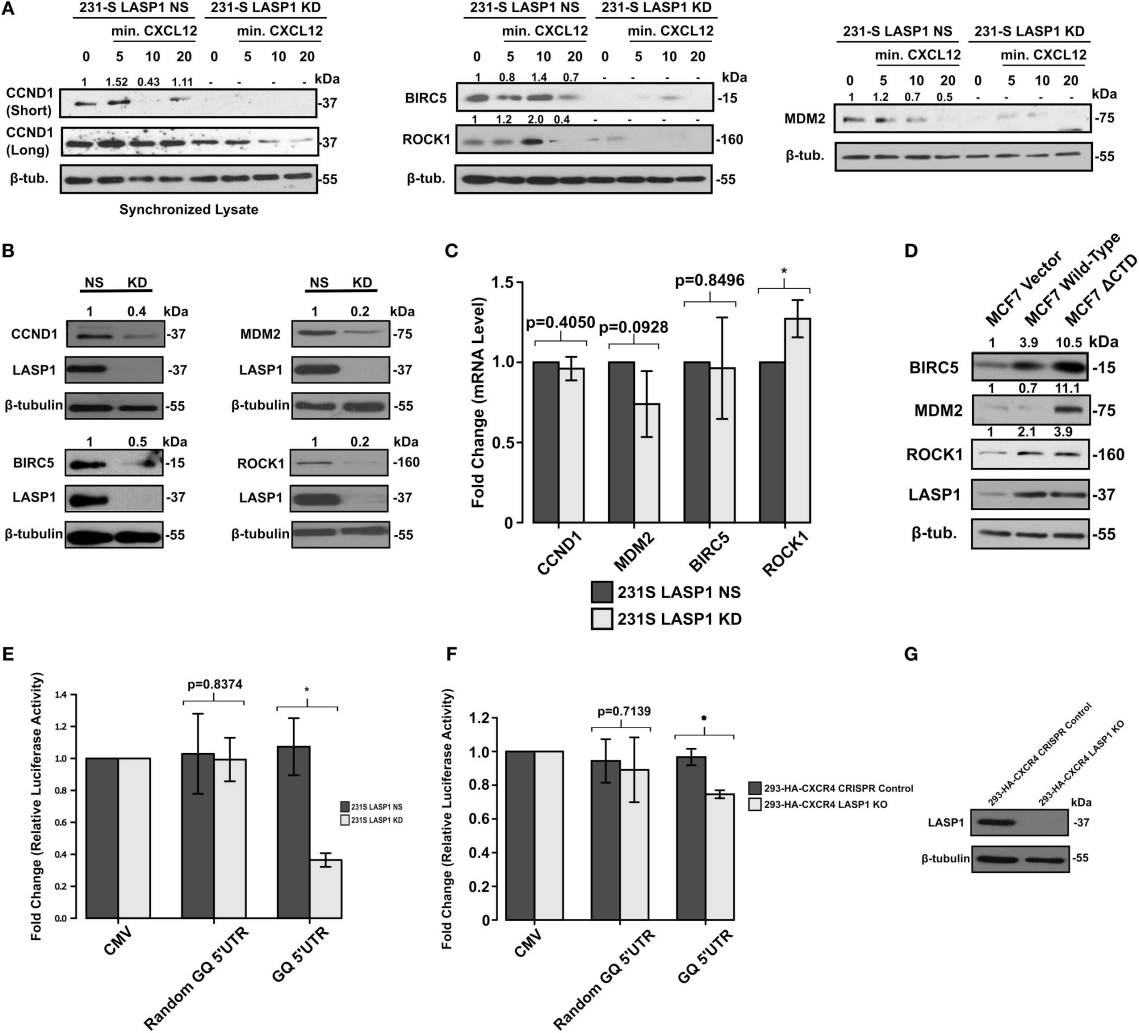
Activation of the CXCR4-LASP1 Axis enhances selective expression of eIF4A-dependent genes. **(A)** 231S LASP1 NS and KD cells were stimulated with 10–20 nM CXCL12. Expression levels of eIF4A-dependent genes were then determined by Western blotting (*n* = 3). Fold change indicates the densitometry ratio of protein signal/β-tubulin with 0 min. set to 1. **(B)** Stable knockdown of LASP1 leads to a reduced expression of eIF4A-dependent genes (*n* = 3). Fold change indicates the densitometry ratio of protein signal/β-tubulin with 231S LASP1 NS cells set to 1. **(C)** Knockdown of LASP1 does not significantly affect CCND1, MDM2, BIRC5, and ROCK1 mRNA levels (*n* = 3). Data was analyzed using the ΔΔCt method with β-tubulin primers as the control. Fold change was calculated with the 231S LASP1 NS cells set to 1. **(D)** Endogenous expression levels of BIRC5, MDM2, and ROCK1 in MCF7 Vector, Wild-type CXCR4, and CXCR4 ΔCTD cells (*n* = 3). Fold change indicates the densitometry ratio of protein signal/β-tubulin with MCF7 vector cells set to 1. **(E)** 231S LASP1 KD cells have a reduced capacity to translate genes harboring a complex 5′UTR as indicated by the GQ 5′UTR luciferase assay (*n* = 3). **(F)** GQ 5′UTR luciferase assay in 293-HA-CXCR4 CRISPR Control and LASP1 KO cells (*n* = 3). Fold change indicates the luminescent ratio of luciferase/renilla (transfection control) with CMV set to 1. **(G)** Western blot analysis of LASP1 protein levels in 293-HA-CXCR4 CRISPR Control and LASP1 KO cells (*n* = 3). ^*^ Indicates *p* < 0.05 as evaluated by unpaired student's *t*-tests.

To determine the functional consequence of CXCR4 and LASP1 on the eIF4F complex, we examined eIF4A-dependent oncogenes commonly associated with cancer. Activation of CXCR4 and the selective expression of cyclin D1 (CCND1), Mdm2, BIRC5, and Rho kinase 1 (ROCK1) in 231S LASP1 NS and KD cells were tested. Stimulation of CXCR4 in control-silenced (LASP1 NS) 231S cells resulted considerable increases in the protein levels of CCND1, BIRC5, Mdm2, and ROCK1 (Figure 6A). On the contrary, when LASP1 is silenced, CXCR4 signaling could not sustain the expression of these proteins downstream of eIF4A1 at comparable control levels.

We then examined the steady state levels of oncogenic proteins that are dependent on the activity of eIF4A in the 231S LASP1 KD cells and serum-starved. Evidently, there was a marked reduction of protein levels of CCND1 (60% reduced), BIRC5 (50% reduced), Mdm2 (80% reduced), and ROCK1 (80% reduced) compared to LASP1 NS cells (Figure 6B). There was no significant reduction in the mRNA levels of these genes as assessed by qPCR to suggest that this difference is occurring at the translational level (Figure 6C). Next, we utilized the MCF7 series to further validate the translational findings of the 231S LASP1 NS/KD cells. As expected, the levels of BIRC5, ROCK1, Mdm2, and including LASP1 itself were increased when CXCR4 was constitutively active (Figure 6D).

To investigate the role of LASP1 in modulating the activity of eIF4A, we employed a synthetic GQ 5′UTR luciferase reporter assay from a documented and validated method (42). This assay allowed us to evaluate the functional activity of eIF4A. Corresponding to the activity of eIF4A, the luciferase activity will either increase or decrease. When LASP1 was stably knocked down, there was a 60% reduction in reporter luciferase activity (less unwinding of GQ 5′UTR) in 231S cells (Figure 6E). This highlights a crucial role of LASP1 in modulating the activity of eIF4A/4B in the eIF4F complex in TNBC cells. To test the effects of LASP1 in other cell types, we generated a KO cell line in 293-HA-CXCR4 cells (Figure 6G). In the 293-HA-CXCR4 LASP1 KO cells, there was a 20% decrease in eIF4A activity (Figure 6F). Taken together, data obtained from the 231S and 293-HA-CXCR4 cells suggests that LASP1 does play a role in modulating the activity of eIF4A. However, the cancer cells are highly reliant on the functional consequence of this interaction.

### Stable Knock Down of LASP1 Sensitizes TNBC Cells to eIF4A Inhibition

**Figure 7:**
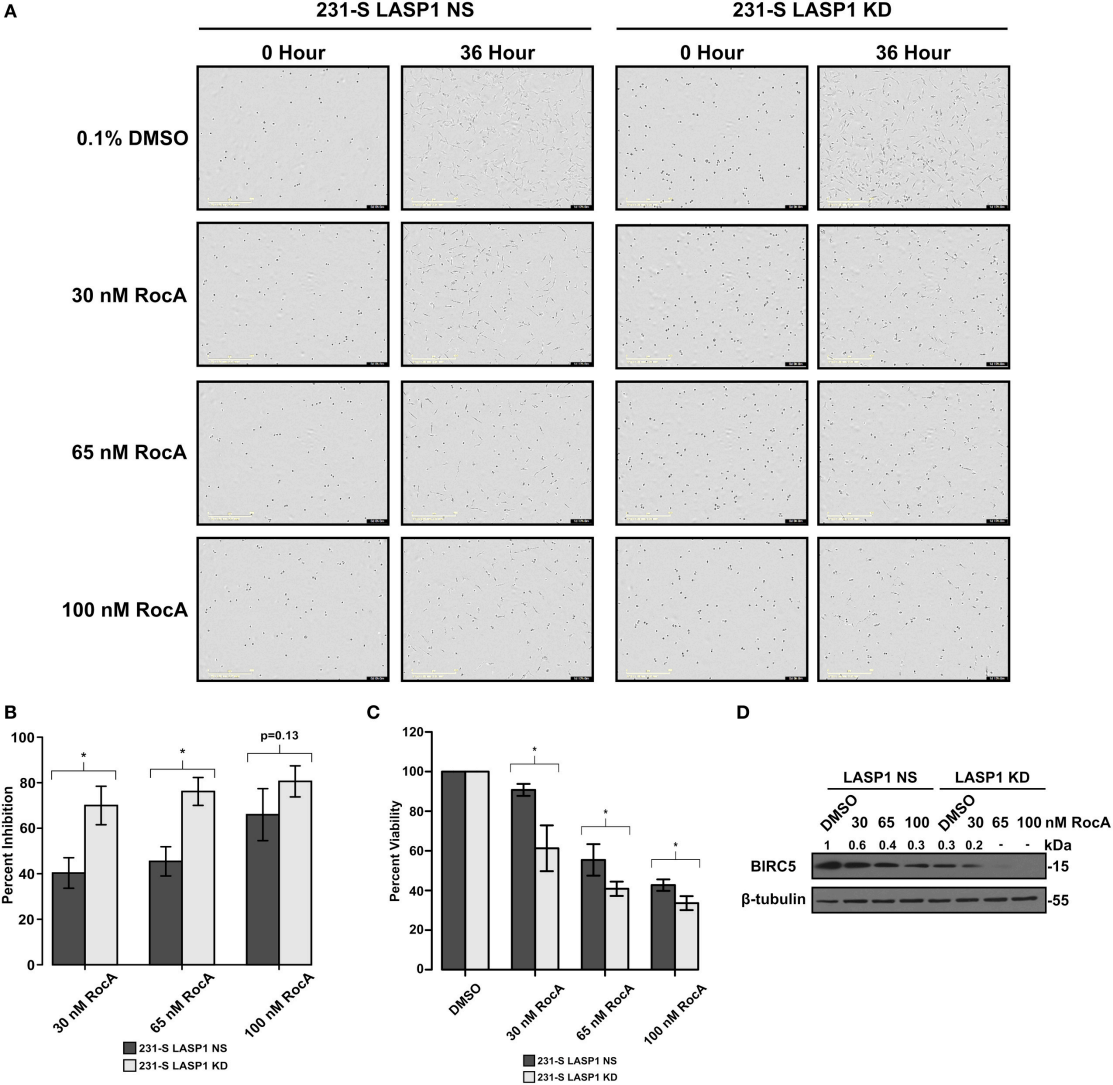
Stable knockdown of LASP1 sensitizes TNBC cells to inhibition by Rocaglamide A. **(A)** Representative images of 231S LASP1 NS and KD cells incubated with various concentrations of Rocaglamide A at 0 and 36-h time points. **(B)** Percent inhibition of 231S LASP1 NS and KD cells following 36-h RocA drug treatment (*n* = 3). Percent inhibition was calculated in reference to the fold difference of percent confluence between Rocaglamide A treated cells and the DMSO control for each cell type at 36 h. **(C)** Percent viability in 231S LASP1 NS and KD cells following Rocaglamide A drug treatment. Data is reflective of absorbance at 450 nm with the DMSO condition set to 100% for each cell type. **(D)** Western blotting of BIRC5 in LASP1 NS/KD cells following 24 h of Rocaglamide A incubation (*n* = 3). Fold change indicates the densitometry ratio of BIRC5 signal/β-tubulin with the 231S LASP1 NS DMSO condition set to 1. ^*^ Indicates *p* < 0.05 as evaluated by student's *t*-tests.

Inhibition of eIF4A has been investigated for its potential as a chemotherapeutic target (28, 38, 58, 59). As such, we examined if the LASP1 deficiency would sensitize 231S TNBC cells to pharmacological inhibition of eIF4A by Rocaglamide A (RocA). Silvestrol, a flavagline family member of Rocaglamide A, was found to have an IC_50_ value of 60 nM in MDA-MB-231 cells (60). We therefore subjected 231S LASP1 NS and KD cells to RocA treatment ranging from 30 to 100 nM (Figure 7A). Stable knock down of LASP1 sensitized the 231S cells to RocA treatment especially at the lowest treatment dose of 30 nM (Figure 7B). Cellular viability also significantly decreased in the LASP1 KD cells with RocA drug treatment (Figure 7C). We verified if RocA inhibited eIF4A, by blotting for levels of BIRC5 protein in LASP1 NS and KD cells. LASP1 NS cells had a dose-dependent decrease in BIRC5 levels. In the LASP1 KD cells, a 70% loss of BIRC5 occurred with LASP1 knock down alone and further decreased with RocA treatment (Figure 7D).

## Discussion

**Figure 8:**
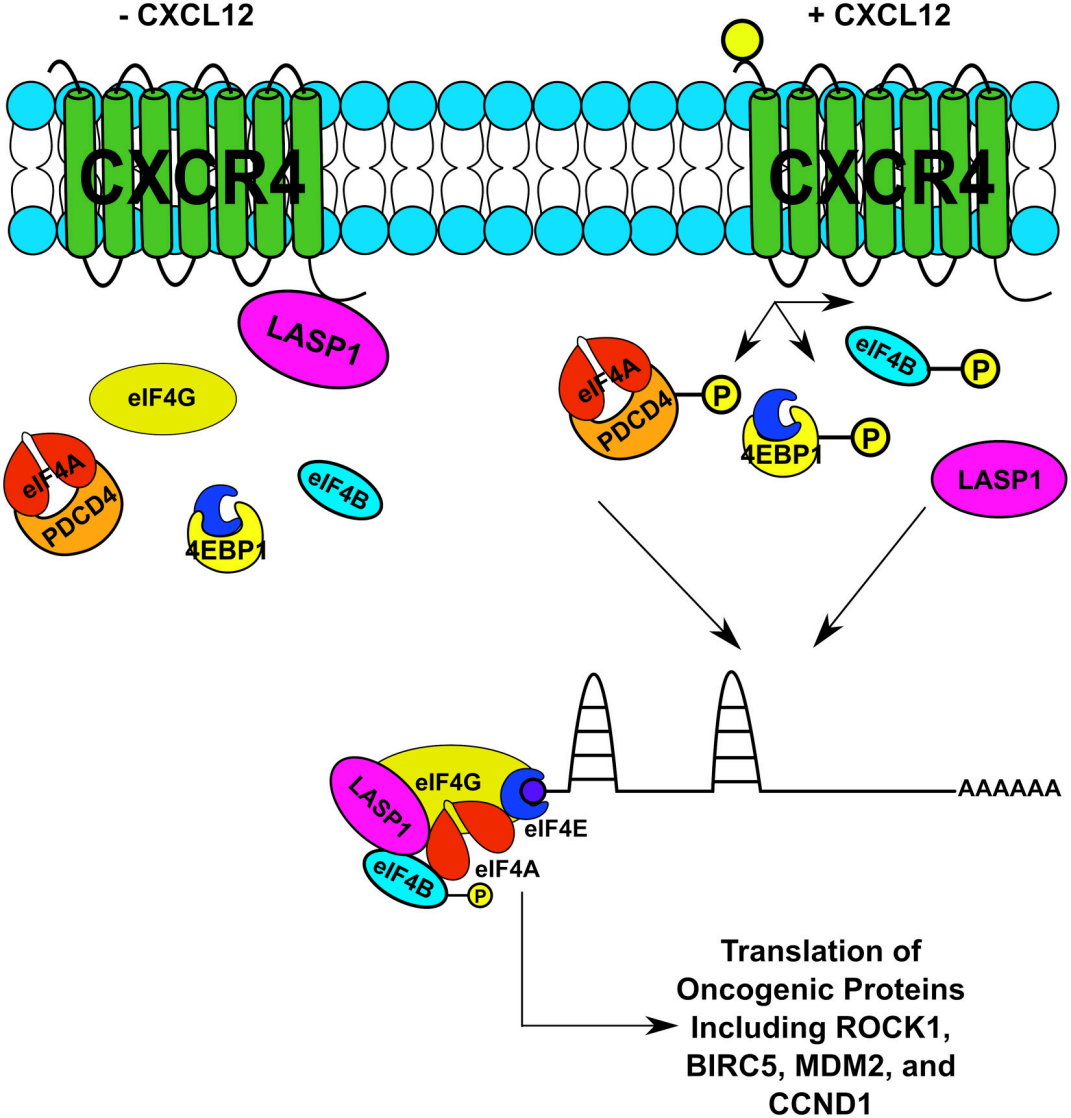
Proposed Model of the CXCR4-LASP1-eIF4F Axis. An illustration of CXCR4 and its relation to the eIF4F complex upon stimulation with CXCL12. This relationship is occurring through two distinctive mechanisms. First, LASP1 dissociates from CXCR4 and directly interacts with eIF4A and eIF4B. Second, phosphorylation of PDCD4, 4E-BP1, and eIF4B is promoted through G protein-coupled receptor signaling. As a result, both complex formation increases along with the function of eIF4A. Consequently, the translation of oncogenic proteins is promoted.

This is the first report that the CXCR4-LASP1 pathway regulates eIF4A1-mediated translation of oncogenic proteins with long and structured 5′UTRs. Based off of our findings, the proposed model is provided (Figure 8). The findings in this study are important as a dysregulation in translational control can rewire the proteome through selective translation of oncogenic mRNAs. The resultant oncoproteins are critical for breast cancer cell survival, tumor progression, local invasion and metastasis (61–66). Protein synthesis is a tightly regulated process. To date, translational initiation has been identified as the rate limiting step. This step of translational regulation is primarily controlled by the eukaryotic initiation 4F complex (eIF4F). In this study, we suggest that CXCR4 can feed into this complex thereby having a significant impact on synthesis of oncogenic proteins needed for breast cancer survival and invasion. To date, there is one additional report suggesting that CXCR4 can influence the protein translational machinery, occurring through an interaction with eIF2B (67). However, the functional consequence of this interaction was not explored in significant detail.

In our initial proteomic screen, eIF4A and eIF4B were identified to interact with LASP1. To confirm this finding, we have provided several pieces of experimental evidence further characterizing this interaction. In the co-immunoprecipitation where eIF4B was immunoprecipitated and blotted for LASP1, three distinctive bands were produced. The one below the LASP1 band (37 kDa) is presumably LASP2 as clone 8C6 anti-LASP1 antibody is known to react with LASP2. However, we hypothesize that the band above 37 kDa is a doubly-phosphorylated form of LASP1 (pY171 and pS146) (68, 69). The human LASP1 that is singly phosphorylated is not reported to shift above 37 kDa thus far. Future studies will need to elucidate the phosphorylation status of LASP1 and the functional consequences interacting with eIF4A and eIF4B. Furthermore, LASP1 associated with eIF4A robustly regardless of the presence or absence of exogenous ATP and Mg^2+^ in the GST-pulldown assay. This may mean that the binding site for LASP1 on the surface of eIF4A is always accessible in spite of conformational changes induced by ATP and Mg^2+^.

Aside from the LASP1 interaction, activation of the CXCR4 pathway led to the formation of the eIF4F complex as evident through several phosphorylation events. First, 4E-BP1 was phosphorylated in a CXCL12-dependent manner similar to the time frame reported in renal cell carcinoma (70). Second, phosphorylation of eIF4B at S422 was similarly observed to increase with CXCL12 stimulation and would affect the rate of translation. Third, an increase in the phosphorylation of PDCD4 following CXCL12 treatment would release eIF4A to incorporate into the eIF4F complex. In all, these three phosphorylation events, in addition to phosphorylation of eIF4E (data not shown), may contribute to active and selective synthesis of oncogenic proteins.

To establish the possibility that LASP1 gets actively recruited into the eIF4F complex upon stimulation with CXCL12, we employed the m^7^-GTP pulldown assay. However, the m^7^-GTP pulldown assay only tells you that components are bound to a complex that “contains” eIF4F. Based off of our findings of the m^7^-GTP experiment and direct binding studies, we hypothesize LASP1 gets actively recruited into the eIF4F complex in a CXCL12-dependent manner. This raises the possibility that LASP1 may assist eIF4A and eIF4B in the unwinding of SLS at the 5′UTR of oncogenic mRNAs. The other key finding of this experiment is the interaction between eIF4G and eIF4E increased in a CXCL12-dependent manner. eIF4G has two binding sites for eIF4A, one of which is necessary for translation and the other plays a modulatory role (71). This brings out the key role played by CXCR4 in enabling the recruitment of eIF4G and LASP1 to enable the synthesis of oncogenic proteins involved in tumor progression and metastasis. It is also interesting to note that LASP1 directly binds to the C-termini of other chemokine receptors including CXCR1, CXCR2, and CXCR3 (16). LASP1 can augmented CXCR2-mediated cell migration (16). Therefore, it is possible that additional chemokine receptors could feed into the eIF4F complex via interactions with LASP1.

If CXCR4 activation led to recruitment of LASP1 and eIF4G into the eIF4F complex and influenced the activity of eIF4A, it would promote the translation of oncogenic mRNAs downstream of eIF4A. As expected, many oncogenic mRNAs with SLS situated at their 5′UTRs including survivin or BIRC5, cyclin D1, Mdm2, and ROCK1 were translated in response to CXCL12 stimulation. These proteins have appreciable roles in breast cancer biology. BIRC5 is involved in cell survival through inhibition of caspase-mediated apoptosis (72). Cyclin D1 is a pivotal protein in the cell cycle. Although nuclear cyclin D1 is known for its role in cell proliferation (73), the cytoplasmic cyclin D1 has a novel, non-canonical role in cell migration (74–76). Cyclin D1 activates CDK4/6 which is a current target in the clinic for chemoresistant cases of breast cancer (77). Next, ROCK1 promotes cell polarization, and persistent directional migration (chemotaxis) (78, 79). Additionally, perturbation of the CXCL12-CXCR4 axis promotes breast cancer cell migration by regulating tumor cell adhesion events through provision of an optimal level of ROCK activity for effective cell migration (80, 81). Finally, Mdm2 has been shown to promote invasive ductal breast carcinoma (IDC) and metastasis and is thought to have additional roles beyond p53 (82). Taking these proteins into account, CXCR4 could therefore have significant (and multifaceted) effects on breast cancer cells through modulation of this translational mechanism.

In summary, we have explored a mechanistic relationship between the CXCR4-LASP1 axis and the regulation of oncoprotein synthesis through specific components of the eIF4F complex. As a result of characterizing this novel protein axis, we hope to provide significant insights in the development of novel small molecule or cell-permeant biopeptide inhibitors. More specifically, inhibiting the interaction between LASP1 and eIF4A may be one approach to sensitize triple-negative breast cancer cells to other inhibitors (23). It is reported that targeting the eIF4F complex may overcome plasticity and heterogeneity issues associated residual disease and chemoresistance (36, 58, 59). This work may facilitate novel modalities of therapy against TNBC breast cancer progression and metastasis (23, 59, 83).

## Author Contributions

CH: designed and executed the experiments, co-wrote the paper. NB: execution of proximity ligation assay. BS: Western blotting in CXCL12 stimulation and signaling experiments. AT: engineering of some DNA constructs. SS: Western blotting in some experiments and intellectual discussion. NV: purification of eIF4A and eIF4B. CF: purification of eIF4A and eIF4B and intellectual discussion. DR: designed experiments, critical and intellectual discussion, co-wrote the paper.

### Conflict of Interest Statement

The authors declare that the research was conducted in the absence of any commercial or financial relationships that could be construed as a potential conflict of interest.
